# The complete mitochondrial genome of the Pale-legged Leaf-Warbler *Phylloscopus tenellipes* (Aves: Phylloscopidae) from Maor Mountain, China

**DOI:** 10.1080/23802359.2022.2100290

**Published:** 2022-07-25

**Authors:** Wei Yu, Shuang Cui, Junda Chen, Dehuai Meng, Xiaoyu Zhou, Liwei Teng, Zhensheng Liu

**Affiliations:** a College of Wildlife and Protected Area, Northeast Forestry University, Harbin, China; b Heilongjiang Province Siberia Tiger Park, Harbin, China; c Key Laboratory of Conservation Biology, National Forestry and Grassland Administration, Harbin, China

**Keywords:** Complete mitochondrial genome, *Phylloscopus tenellipes*, Leaf-Warbler

## Abstract

The Pale-legged Leaf-Warbler (*Phylloscopus tenellipes*) (Swinhoe, 1860) is an olive-brown warbler that is extensively dispersed in Asia. We sequenced the whole mitochondrial genome of a Pale-legged Leaf-Warbler collected on Maor Mountain, Heilongjiang Province, China. The mitochondrial genome is 16,972 bp and contains 13 protein-coding genes, two rRNA genes, 22 tRNA genes, and one control region (CR). The CR is 1098 bp. The nucleotide sequence is composed of 29.11% A, 22.98% T, 14.64% G, and 33.27% C. Phylogenetic research revealed that *P. tenellipes* is closely related to *Phylloscopus borealoides*.

The Pale-legged Leaf-Warbler (*Phylloscopus tenellipes*) (Swinhoe, 1860), commonly known as the Pale-legged Warbler, is a small olive-brown warbler (Otani 2013). *P. tenellipes* breeds in East Asia and spends the winter in Myanmar, the Indochina Peninsula, and the Malay Peninsula, as well as passing through most provinces in eastern China (Hungnon et al. 2017). It lives in forest and shrub areas, mostly on the ground, and feeds on insects (Riegner 1998; Fujimaki 2011). This species was classified as Least Concern on the Red List because it has a very large range and the population trend appears to be stable (Xiong et al. 2006; Xia and Lin 2011).

*P. tenellipes*' complete mitochondrial genome was sequenced using muscle tissue collected from Maor Mountain in China (127°30′-127°34′E, 45°20′-45°25′N). The specimens were deposited at the College of Wildlife and Protected Area of Northeast Forestry University under the voucher number DJLY202106 (Zhensheng Liu, Email: zhenshengliu@163.com). To undertake paired-end (PE) sequencing, we used the whole-genome shotgun (WGS) method and next-generation sequencing (NGS) based on the Illumina HiSeq sequencing technology. The sequence was submitted to GenBank with the accession number OL628872.

The mitochondrial genome consists of 13 protein-coding genes (PCGs), two rRNA genes (12S rRNA and 16S rRNA), 22 tRNA genes, and one control region (CR). The genome is 16,972 bp in total length and the total sequence GC content is 47.91%, with a base composition of 29.11% A, 22.98% T, 14.64% G, and 33.27% C. The total length of 13 protein-coding genes is 11,426 bp long and begins with ATG (ND1, ND2, ND3, ND4, ND4L, ND5, ND6, COX1, COX2, COX3, ATP6, ATP8, and CYTB). The total length of all tRNA genes is 1562 bp long, ranging from 42 bp (tRNA-Ile) to 70 bp (tRNA-Gln). The lengths of the two rRNA genes are 978 bp (12s rRNA) and 1601 bp (16s rRNA), respectively, with a control region of 1098 bp.

The alignment method of DNA spectrograms can be used to facilitate analysis of very long sequences or entire genomes at different resolutions. To infer phylogenetic relationships, we used MEGA7 (Kumar et al. 2016) and the maximum-likelihood method (Wen et al. 2017). The bootstrap consensus tree inferred from 1000 replicates is taken to represent the evolutionary history of the taxa analyzed (Felsenstein 1985). As shown in the phylogenetic tree (Figure 1), the phylogenetic relationship of the Pale-legged Leaf-Warbler is very close to that of *Phylloscopus borealoides* (MN125373.1).

**Figure 1.**
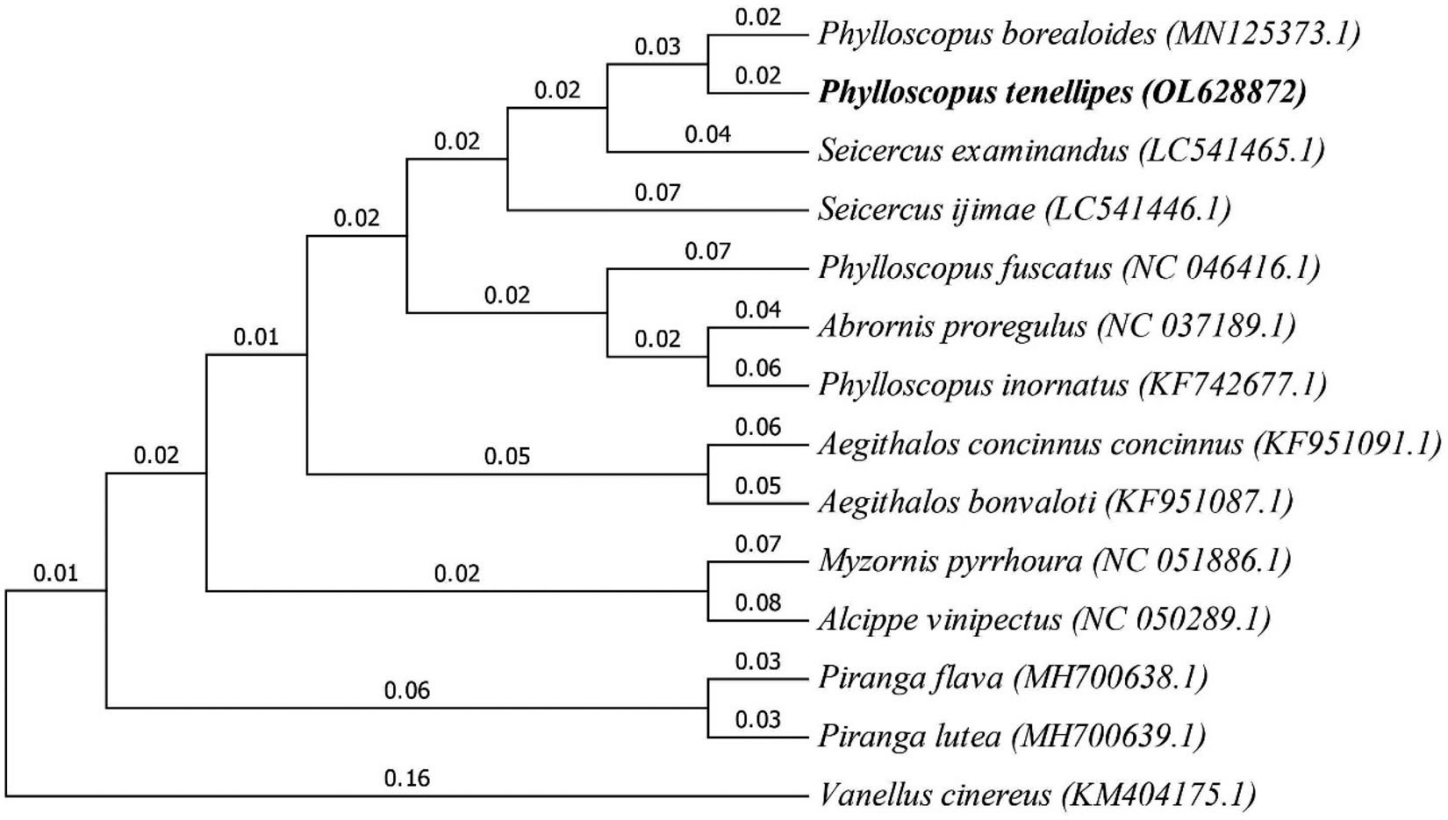
Phylogenetic tree generated using the maximum-likelihood method based on complete mitochondrial genomes of *P. tenellipes* and 13 other closely species.

## Ethical approval

The muscular tissue of a Pale-legged Leaf-Warbler was removed from an individual that perished in the wild at the age of three days. The first day of searching in Maor Mountain yielded no results, while the second yielded a body. In this scenario, ethical clearance is not necessary.

## Author contributions

We would like to express our gratitude to Professors Zhensheng Liu, Liwei Teng, and Xiaoyu Zhou for their contributions to the study's concept and design; we also thank Dr. Junda Chen for teaching us to analyze our data. Dr. Shuang Cui and Dr. Dehuai Meng were in charge of gathering samples and reviewing the work for scientific substance. All authors agree to be solely responsible for their contributions.

## Data Availability

The data supporting this study's findings are freely available in NCBI at https://www.ncbi.nlm.nih.gov/, reference number OL628872. The associated BioProject, SRA, and Bio-Sample numbers are PRJNA782249, SRR16996949, and SAMN23371668, respectively.
